# Application of Artificial Intelligence to Plasma Metabolomics Profiles to Predict Response to Neoadjuvant Chemotherapy in Triple-Negative Breast Cancer

**DOI:** 10.3389/frai.2022.876100

**Published:** 2022-08-11

**Authors:** Ehsan Irajizad, Ranran Wu, Jody Vykoukal, Eunice Murage, Rachelle Spencer, Jennifer B. Dennison, Stacy Moulder, Elizabeth Ravenberg, Bora Lim, Jennifer Litton, Debu Tripathym, Vicente Valero, Senthil Damodaran, Gaiane M. Rauch, Beatriz Adrada, Rosalind Candelaria, Jason B. White, Abenaa Brewster, Banu Arun, James P. Long, Kim Anh Do, Sam Hanash, Johannes F. Fahrmann

**Affiliations:** ^1^Department of Biostatistics, The University of Texas MD Anderson Cancer Center, Houston, TX, United States; ^2^Department of Clinical Cancer Prevention, The University of Texas MD Anderson Cancer Center, Houston, TX, United States; ^3^Department of Breast Medical Oncology, The University of Texas MD Anderson Cancer Center, Houston, TX, United States; ^4^Breast Cancer Research Program, Baylor College of Medicine, Houston, TX, United States; ^5^Department of Abdominal Imaging, The University of Texas MD Anderson Cancer Center, Houston, TX, United States; ^6^Department of Breast Imaging, The University of Texas MD Anderson Cancer Center, Houston, TX, United States

**Keywords:** triple-negative breast cancer, biomarkers, artificial intelligence, deep-learning model, neoadjuvant chemotherapy, prediction

## Abstract

There is a need to identify biomarkers predictive of response to neoadjuvant chemotherapy (NACT) in triple-negative breast cancer (TNBC). We previously obtained evidence that a polyamine signature in the blood is associated with TNBC development and progression. In this study, we evaluated whether plasma polyamines and other metabolites may identify TNBC patients who are less likely to respond to NACT. Pre-treatment plasma levels of acetylated polyamines were elevated in TNBC patients that had moderate to extensive tumor burden (RCB-II/III) following NACT compared to those that achieved a complete pathological response (pCR/RCB-0) or had minimal residual disease (RCB-I). We further applied artificial intelligence to comprehensive metabolic profiles to identify additional metabolites associated with treatment response. Using a deep learning model (DLM), a metabolite panel consisting of two polyamines as well as nine additional metabolites was developed for improved prediction of RCB-II/III. The DLM has potential clinical value for identifying TNBC patients who are unlikely to respond to NACT and who may benefit from other treatment modalities.

## Introduction

Triple-negative breast cancer (TNBC) accounts for ~15–20% of breast cancers and represents a heterogeneous subtype characterized by high pathological grade, strong invasiveness, local recurrence, high metastasis rate, and poor prognosis (Foulkes et al., 2010). TNBCs are defined based on the lack expression of estrogen receptor (ER), progesterone receptor (PR), and human epidermal growth factor receptor type 2 (HER2) and are thus not amenable to endocrine therapy or therapies targeted to the HER2 receptor type (Foulkes et al., 2010). Chemotherapy remains the mainstay of systemic treatment, typically consisting of anthracycline and taxane-based chemotherapy regimens (Foulkes et al., 2010; Bianchini et al., 2022). Platinum-based neoadjuvant chemotherapy has been shown to increase pathological complete response (pCR) rates compared to platinum-free neoadjuvant chemotherapy. However, platinum-based treatment is associated with higher rates of toxicity and treatment discontinuation, and the optimal integration of platinum-based agents remains controversial (Poggio et al., 2018). The addition of immunotherapy has shown promise with recent Phase III clinical trials demonstrating that the addition of the anti-PD-L1 inhibitor atezolizumab or the anti-PD1 inhibitor pembrolizumab with chemotherapy improved pCR compared to chemotherapy alone in patients with TNBC (Schmid et al., 2018, 2020).

In the curative neoadjuvant setting, a pCR after neoadjuvant chemotherapy (NACT) in TNBC is associated with improved long-term survival yielding estimated 10-year relapse survival rates of 86% (Symmans et al., 2017). However, up to 60% of patients will have residual disease after receiving standard NACT and are at an elevated risk of poor outcome, with reported 10-year estimated relapse survival rates of 81, 55, and 23% for TNBC patients with a residual cancer burden (RCB) index of I, II, and III, respectively (Huober et al., 2010; Symmans et al., 2017; Schmid et al., 2020). Currently, there is a paucity of biomarkers that can reliably identify TNBC patients that will have poor response to NACT.

Polyamines, including putrescine, spermidine, and spermine, are polycationic alkylamines that are essential for eukaryotic cell growth. Dysregulation of polyamine metabolism is frequent in cancer and polyamines have been reported to play central roles in neoplastic transformation and tumor progression (Park and Igarashi, 2013; Casero et al., 2018; Chia et al., 2022). We previously obtained evidence that increased plasma levels of the acetylated polyamine diacetylspermine (DAS) in TNBC was prognostic for poor progression-free survival and overall survival. Specifically, we found that elevated levels of plasma DAS to be prognostic for worse 5-year metastasis free survival and poor 5-year overall survival in newly-diagnosed treatment naïve TNBC patients (Fahrmann et al., 2020).

Here, we tested the utility of plasma polyamines for identifying subjects who will be insensitive to NACT as part of a comprehensive plasma metabolomics profiling. We further applied artificial intelligence to plasma metabolic profiles and, using a deep-learning model (DLM), established a metabolite biomarker panel consisting of two polyamines as well as nine additional metabolites for prediction of response to NACT.

## Materials and Methods

### Specimen Sets

Patients with stage I–III TNBC enrolled in the prospective, Institutional Review Board (IRB)-approved, clinical study, “A Robust TNBC Evaluation framework to Improve Survival” (ARTEMIS, NCT02276443), were included in this study. Briefly, the ARTEMIS trial included treatment-naïve patients with localized TNBC (stage I-III) that underwent a pre-treatment ultrasound with biopsy following by 4 cycles of Adriamycin-cyclophosphamide (AC) chemotherapy. The outcome of the molecular characterization from the pre-treatment biopsy in combination with response assessment (clinical exam/diagnostic imaging, after 4 cycles of AC) were used to identify chemotherapy-insensitive disease and to inform the second phase of neoadjuvant therapy. Patients deemed to have chemo-sensitive disease after 4 cycles of AC (≥70% volumetric reduction by ultrasound after 4 cycles of AC) were recommended to undergo standard paclitaxel-based chemotherapy as the second phase of their NACT consisting of 4 cycles or weekly for 12 doses. Patients with TNBC predicted to be chemo-insensitive (≤ 70% volumetric reduction by ultrasound after 4 cycles of AC) were offered therapy on clinical trials using targeted therapy in combination with chemotherapy based on the specific molecular characteristics of their tumor as the second phase of their therapy with dose regimens varying depending on therapy. Response to neoadjuvant therapy was determined using the residual cancer burden (RCB) index (Symmans et al., 2007). The specimen set consisted of pre-treatment EDTA plasma from 88 patients who received standard-of-care NACT; 62 of the 88 patients had a second plasma sample available after four cycles of AC. Detailed patient and tumor characteristics are provided in Table 1.

**Table 1:** Patient and tumor characteristics.

	**TNBC^†^ cases**	**Controls**
N	88	167
Age, mean +/– SD	50 +/– 11	58 +/– 9
Stage, *N* (%)		
I	9 (10)	–
II	64 (73)	–
III	15 (17)	–
RCB status, *N* (%)		
0	48 (55)	–
I	14 (16)	–
II	21 (24)	–
III	5 (6)	–

*†All TNBC patients received NACT; plasma samples were collected pre-treatment*.

*TNBC, triple-negative breast cancer; RCB, Residual Cancer Burden*.

EDTA plasma from cancer-free women (*n* = 167) were obtained from the MD Anderson Cancer Center (MDACC) Longitudinal High-Risk Cohort initiated September 1st, 2011, for the prospective follow-up of cancer-free high-risk women seen in the MDACC Cancer Prevention Center (IRB protocol LAB07-0086).

### Immunohistochemistry

Immunohistochemical (IHC) staining for Ki-67 was performed on unstained 4-μm-thick tissue sections that had been prepared from a representative paraffin block of tumor in each case. IHC staining for Ki-67 was performed using the polymeric biotin-free horseradish peroxidase method on the Leica Microsystems Bond III autostainer (Leica Microsystems, Buffalo Grove, IL, USA). The slides were incubated at 60°C for 25 min. Following heat-induced epitope retrieval with Tris-EDTA buffer for 20 min at 100°C, slides were incubated with mouse monoclonal antibody to Ki-67 (clone MIB-1, Dako; 1:100). The Refine Polymer Detection kit was used to detect bound antibody, with 3,3-diaminobenzidine serving as the chromogen (Leica Microsystems). For Ki-67, the percentage of any nuclear staining of any intensity in the tumor cells was recorded.

### Metabolomic Analysis

#### Sample Preparation

Plasma metabolites were extracted from pre-aliquoted biospecimens (15 μL) with 45 μL of LCMS grade methanol (ThermoFisher) in a 96-well microplate (Eppendorf). Plates were heat sealed, vortexed for 5 min at 750 rpm, and centrifuged at 2,000 × g for 10 mins at room temperature. The supernatant (30 μL) was transferred to a 96-well plate, leaving behind the precipitated protein. The supernatant was further diluted with 60 μL of 100 mm ammonium formate, pH3 (Fisher Scientific). For Hydrophilic Interaction Liquid Chromatography (HILIC) positive ion analysis, 15 μL of the supernatant and ammonium formate mix were diluted with 195 μL of 1:3:8:144 water (GenPure ultrapure water system, Thermofisher): LCMS grade methanol (ThermoFisher): 100 mm ammonium formate, pH3 (Fisher Scientific): LCMS grade acetonitrile (ThermoFisher). For C18 analysis, 15 μL of the supernatant and ammonium formate mix were diluted with 90 μL water (GenPure ultrapure water system, ThermoFisher) for positive ion mode. Each sample solution was transferred to 384-well microplate (Eppendorf) for LCMS analysis.

#### Untargeted Analysis of Primary Metabolites and Biogenic Amines

Untargeted metabolomics analysis was conducted on Waters Acquity™ UPLC system with 2D column regeneration configuration (I-class and H-class) coupled to a Xevo G2-XS quadrupole time-of-flight (qTOF) mass spectrometer as previously described (Fahrmann et al., 2019, 2020, 2021a,b). Chromatographic separation was performed using HILIC (Acquity™ UPLC BEH amide, 100 Å, 1.7 μm 2.1 × 100 mm, Waters Corporation, Milford, U.S.A) and C18 (Acquity™ UPLC HSS T3, 100 Å, 1.8 μm, 2.1 × 100 mm, Water Corporation, Milford, U.S.A) columns at 45°C.

Quaternary solvent system mobile phases were (A) 0.1% formic acid in water, (B) 0.1% formic acid in acetonitrile and (D) 100 mm ammonium formate, pH 3. Samples were separated on the HILIC using the following gradient profile at 0.4 mL/min flow rate: (95% B, 5% D) linear change to (70% A, 25% B and 5% D) over 5 min; 100% A for 1 min; and 100% A for 1 min. For C18 separation, the chromatography gradient was as follows at 0.4 mL/min flow rate: 100% A with a linear change to (5% A, 95% B) over 5 min; (95% B, 5% D) for 1 min; and 1 min at (95% B, 5% D).

A binary pump was used for column regeneration and equilibration. The solvent system mobile phases were (A1) 100 mm ammonium formate, pH 3, (A2) 0.1% formic in 2-propanol and (B1) 0.1% formic acid in acetonitrile. The HILIC column was stripped using 90% A2 for 5 min at 0.25 mL/min flow rate, followed by a 2 min equilibration using 100% B1 at 0.3 mL/min flow rate. Reverse phase C18 column regeneration was performed using 95% A1, 5% B1 for 2 min followed by column equilibration using 5% A1, 95% B1 for 5 min at 0.4 mL/min flow rate.

#### Mass Spectrometry Data Acquisition

Mass spectrometry data was acquired using ‘sensitivity' mode in positive and negative electrospray ionization mode within 50–800 Da range. For the electrospray acquisition, the capillary voltage was set at 1.5 kV (positive), sample cone voltage 30 V, source temperature at 120°C, cone gas flow 50 L/h and desolvation gas flow rate of 800 L/h with scan time of 0.5 sec in continuum mode. Leucine Enkephalin; 556.2771 Da (positive) was used for lockspray correction and scans were performed at 0.5 sec. The injection volume for each sample was 6 μL. The acquisition was carried out with instrument auto gain control to optimize instrument sensitivity over the samples acquisition time.

Data were processed using Progenesis QI (Non-linear, Waters). Peak picking and retention time alignment of LC-MS and MSe data were performed using Progenesis QI software (Non-linear, Waters). Data processing and peak annotations were performed using an in-house automated pipeline as previously described (Fahrmann et al., 2019, 2020, 2021a; Vykoukal et al., 2020). Annotations were determined by matching accurate mass and retention times using customized libraries created from authentic standards and by matching experimental tandem mass spectrometry data against the NIST MSMS, LipidBlast or HMDB v3 theoretical fragmentations. To correct for injection order drift, each feature was normalized using data from repeat injections of quality control samples collected every 10 injections throughout the run sequence. Measurement data were smoothed by Locally Weighted Scatterplot Smoothing (LOESS) signal correction (QC-RLSC) as previously described. Values are reported as ratios relative to the median of historical quality control reference samples run with every analytical batch for the given analyte (Fahrmann et al., 2019, 2020, 2021a; Vykoukal et al., 2020).

### Statistical Analysis

A deep learning algorithm employing all quantified metabolites with tuned hyperparameters using the grid search approach (Candel et al., 2016) was run 20 times, and the relevance importance score for each metabolite was calculated using the Gedeon method (Gedeon, 1997). Metabolites were prioritized based on consistently exhibiting a relative importance score >0.5. Ten models, including deep learning, random forest, ensemble learning and gradient boosting method algorithms, incorporating eleven metabolites were assessed for distinguishing responder/partial responders (RCB-0/I) from non-responders (RCB-II/III). The predictability, reliability, and stability of the models in the training set was evaluated using a 5-fold cross validation as well as through introducing perturbations (e.g., random sample selection with replacement) to the dataset.

Model discrimination was assessed based on receiver operating characteristic curve (ROC), as well as sensitivity and specificity estimates. The 95% confidence intervals (CI) for AUCs were estimated using the Delong method (DeLong et al., 1988). All modeling was performed using the H_2_O package and R statistical program (Candel et al., 2016).

## Results

### Plasma Polyamine Levels in Triple-Negative Breast Cancer

**Figure 1:**
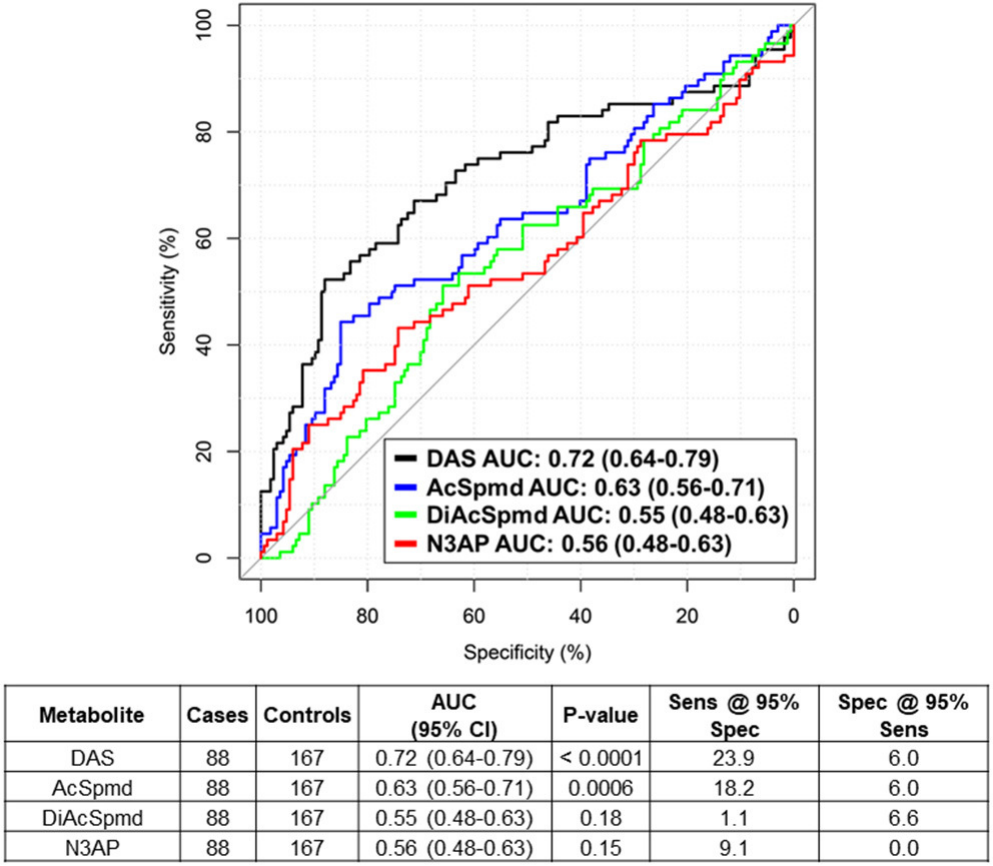
Predictive performance of individual polyamines for distinguishing treatment-naïve TNBC cases from healthy controls. Table beneath shows AUC (95% CI), Wilcoxon rank sum test 2-sided *P*-values as well as sensitivity and specificity estimates at 95% specificity/sensitivity thresholds of individual polyamines for distinguishing TNBC cases from healthy controls.

Using mass spectrometry, we first assessed polyamines levels in plasmas from 88 newly diagnosed treatment-naïve TNBC cases and 167 cancer-free women enrolled in the MDACC Longitudinal High-Risk Cohort (Table 1). A total of four polyamines, acetylspermidine (AcSpmd), diacetylspermidine (DiAcSpmd), diacetylspermine (DAS), and N-(3-acetamidopropyl)pyrrolidin-2-one (N3AP) were detected and quantified. Of these, AcSpmd and DAS were statistically significantly elevated (Wilcoxon rank sum test 2-sided *p* < 0.01) in case plasmas compared to controls (Figure 1). DAS exhibited the highest discrimination performance for distinguishing all cases from controls with an AUC of 0.72 (95% C.I.: 0.64–0.79) (Figure 1).

### Association of Polyamines With RCB Status

All 88 TNBC patients were treated with AC in the neoadjuvant setting. A subset of 62 (70.5%) had a complete pathological response (pCR/RCB-0) or minimal residual disease (RCB-I) following NACT, whereas 26 (29.5%) had a moderate to extensive tumor burden (RCB-II and III) (Table 1). Pathological response tended to be associated with tumor stage and % tumoral Ki-67 staining positivity, albeit not statistically significant (Supplementary Figure 1).

Elevated pre-treatment plasma levels of AcSpmd, N3AP, DiAcSpmd and DAS were associated with higher odds of RCB-II/III following NACT [adjusted ORs of 1.24 (95% CI: 0.76–2.04), 1.33 (95% CI: 0.79–2.46), 1.15 (95% CI: 0.71–1.85) and 1.26 (95% CI: 0.71–1.91) per standard deviation increase, respectively] (Table 2).

**Table 2:** Performance estimates of polyamines for distinguishing RCB-II/III from RCB-0/I.

**Polyamines**	**AUC (95% CI)**	**Sensitivity** **@ 95% sen**	**Specificity @ 95% spec**	**Odds ratio**	**Adjusted Odds ratio^†^**
AcSpmd	0.59 (0.46–0.71)	0.12 (0.00–0.23)	0.19 (0.10–0.31)	1.34 (0.85–2.10)	1.24 (0.76–2.04)
N3AP	0.55 (0.42–0.68)	0.12 (0.00–0.27)	0.10 (0.02–0.32)	1.34 (0.86–2.26)	1.33 (0.79–2.46)
DiAcSpmd	0.54 (0.40–0.67)	0.08 (0.00–0.23)	0.08 (0.00–0.26)	1.15 (0.72–1.80)	1.15 (0.71–1.85)
DAS	0.58 (0.46–0.71)	0.15 (0.00–0.31)	0.24 (0.10–0.39)	1.39 (0.89–2.23)	1.26 (0.77–2.10)

*Area under the Receiver Operating Characteristic Curve (AUC), sensitivity, specificity, odds ratios, and adjusted odds ratios estimates and corresponding 95% confidence intervals of individual polyamines are shown. AcSpmd, acetylspermidine; N3AP, N-(3-acetamidopropyl)pyrrolidin-2-one; DiAcSpmd, diacetylspermidine; DAS, diacetylspermine.^†^age and stage were included as covariables in adjusted odd ratios*.

### Applying Artificial Intelligence to Metabolic Profiles to Develop a Combination Rule for Prediction of RCB-II/III

Complementary to the four polyamines, untargeted metabolomics analyses of these plasmas yielded an additional 82 uniquely annotated metabolite features (Supplementary Table 1). To prioritize metabolites associated with response to NACT for model building, relative importance scores were calculated using the Gedeon method (Gedeon, 1997) and metabolites were selected that constantly showed a relative important score of > 0.5 (*see Methods*). This approach resulted in 11 cancer-related metabolites, consisting of two polyamines, two lipids, three amino acids, a purine catabolite, and two indole-derivatives (Supplementary Table 2). Spearman correlation analyses indicated low to moderate associations between these metabolites (Supplementary Figure 2).

**Figure 2:**
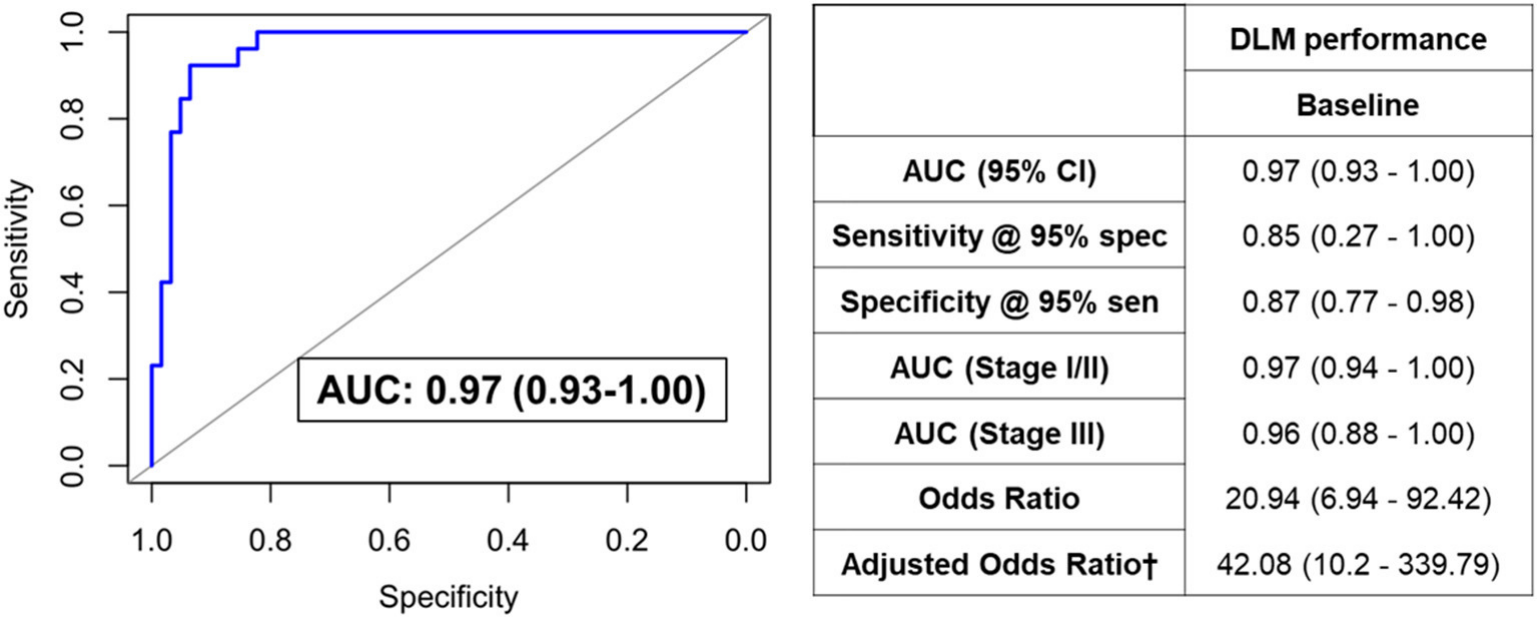
ROC curve for the DLM for distinguishing TNBC patients that went on to have RCB-II/III following NACT from those that had RCB-0/I. Table provides tabulated performance estimates of the DLM.^†^Age and stage were included as covariables in adjusted odd ratios.

We next sought to develop a machine learning algorithm that incorporated the eleven metabolites for predicting RCB-II/III. For model building, we tested 10 different machine learning algorithms (Table 3). Of these, a deep learning model (DLM) with 3 hidden layers and 20 nodes in each layer achieved the highest predictive performance with an AUC of 0.97 (95% CI: 0.93–1.00) with 85% sensitivity at 95% specificity for identifying RCB-II/III (Figure 2). Notably, the DLM yielded an AUC of 0.76 (95% CI: 0.65–0.87) with 48% sensitivity at 95% specificity for distinguishing TNBC cases with residual disease (RCB-I/II/III) from those that achieved a pCR (Supplementary Table 3). To assess model reproducibility and stability, we introduced perturbation into the dataset (e.g., random selection with replacement) and re-evaluated model performance, the results of which showed that the DLM was robust (Supplementary Table 4).

**Table 3:** Performance of the different learning models in the training set.

**Model**	**Hyper parameters**	**AUC**	**Log loss**	**AUCpr**	**Mean per class error**	**RMSE**
Deep learning model	Activation: Maxout, hidden layers:3, number of nodes in each layer: 20	0.97	0.396	0.62	0.249	0.339
Deep learning model	Activation: Maxout, hidden layers:2, number of nodes in each layer = 1	0.86	0.412	0.61	0.268	0.385
Deep learning model	Activation: Tanh, hidden layers: 1, number of nodes in each layer = 3	0.78	0.429	0.60	0.283	0.393
Deep learning model	Activation: Tanh hidden layers:1, number of nodes in each layer: 1	0.72	0.438	0.60	0.297	0.399
GLM	Family: Binomial	0.68	0.585	0.53	0.331	0.47
Gradient boosting method	Number of tree: 50, Maximum depth:6	0.61	0.692	0.53	0.342	0.499
Distributed random forest (DRF)	–	0.55	0.709	0.51	0.49	0.507
Extremely randomized trees (XRT)	–	0.53	0.787	0.45	0.429	0.537
StackedEnsemble	Ensemble models (best of each family): GLM, Deep Learning, Random Forest, Gradient Boost Method	0.53	2.274	0.46	0.421	0.671
Extreme gradient boosting	–	0.52	4.198	0.47	0.481	0.66

*AUC, Area under the ROC curve; AUCpr, Area under precision-recall curve; RMSE, root-mean-square deviation; GLM, generalized linear model; DRF, Distributed Random Forest; XRT, Extremely Randomized Trees*.

**Figure 3:**
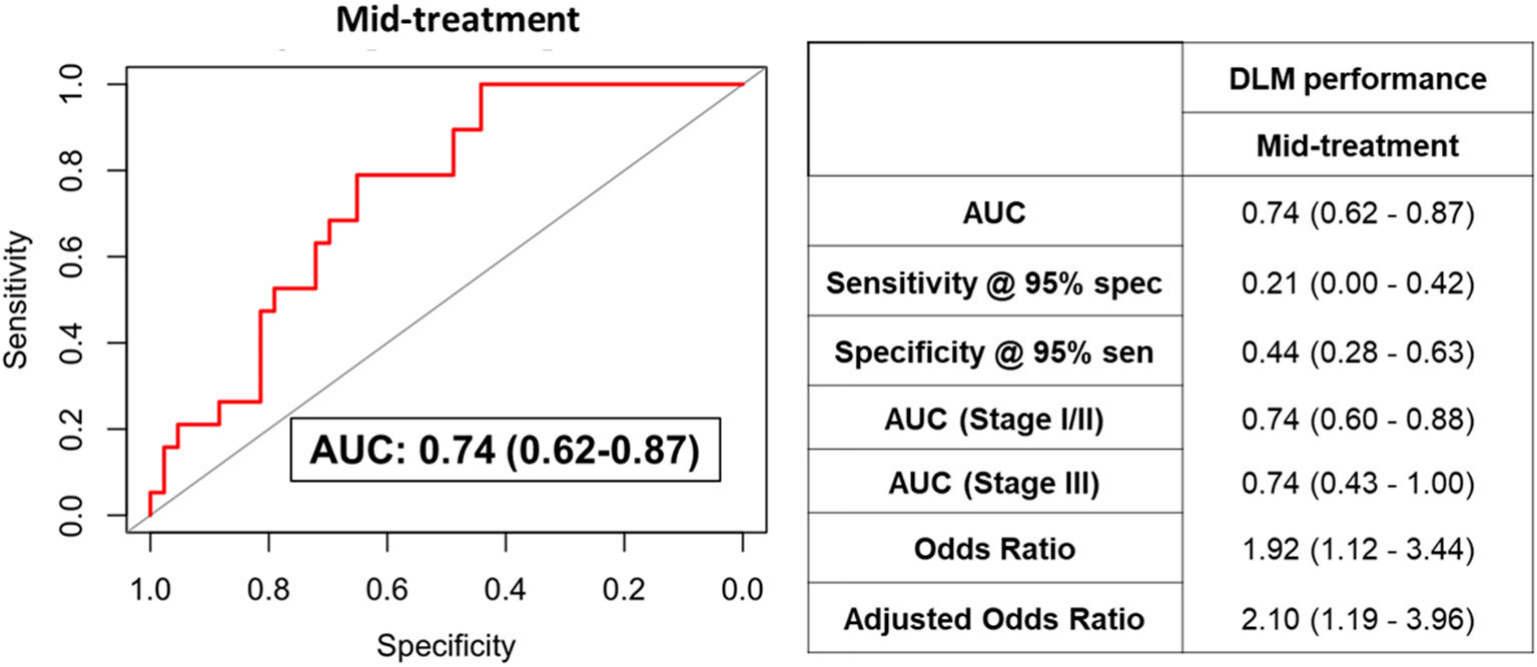
ROC curve for the DLM for distinguishing TNBC patients that went on to have RCB-II/III following NACT from those that had RCB-0/I using plasmas collected after four cycles of AC. Table provides tabulated performance estimates of the DLM.^†^Age and stage were included as covariables in adjusted odd ratios.

We additionally assessed the predictive performance of the DLM model using plasma samples collected during NACT from a subset of TNBC patients (*n* = 62). The DLM model showed an AUC of 0.74 (95% CI: 0.62–0.87) with 21% sensitivity at 95% specificity for RCB-II/III (Figure 3).

## Discussion

The heterogeneity of TNBC results in a spectrum of responses to NACT with pCR being achieved in only a subset of patients (Sikov et al., 2015; Gamucci et al., 2018). Several methods have been used to measure and predict residual disease during course of treatment, including ultrasound, MRI scans, histopathology; however, none have yet achieved adequate performance to predict response to NACT (Croshaw et al., 2011; Shin et al., 2011; Ono et al., 2012; De Los Santos et al., 2013; Leon-Ferre et al., 2018). Here, we applied artificial intelligence to metabolomic profiles of TNBC patient plasmas obtained prior to NACT and, using a DLM, establish a blood-based polyamine-centric metabolite panel that is predictive of non-response to NACT. The metabolite panel may be implemented in the clinical setting to stratify TNBC patients who are at high-risk of being non-responsive to NACT and who may benefit from alternate treatment modalities. Conversely, TNBC patients who are likely to be responsive to NACT may potentially benefit from dose de-escalation, thereby permitting management of treatment-associated toxicity.

The metabolite panel consisted of several cancer-relevant metabolites including the acetylated polyamines DAS and AcSpmd, which were found to be elevated in TNBC patients who were less likely to respond to NACT. Elevated levels of acetylated polyamines in various biofluids including urine, plasma, and serum, have been shown to report on cancer status (Park and Igarashi, 2013; Wikoff et al., 2015; Fahrmann et al., 2019, 2020, 2021b). Targeting of cancer cell polyamine metabolism *via* small molecule inhibitors has been proposed for anti-cancer therapy for cancer, including TNBC (Casero et al., 2018; Geck et al., 2020; Capellen et al., 2021). Acetylation of polyamines is mediated by spermidine/spermine N1-acetyltransferase 1 (SAT1) (Pegg, 2013; Fahrmann et al., 2020). Our prior investigations demonstrated that oncogenic MYC regulates transcription of polyamine metabolizing enzymes ornithine decarboxylase (ODC1), spermidine synthase (SRM), and spermine synthase (SMS) in TNBC, and that elevated intracellular polyamine levels induce expression of SAT1 (Pegg, 2013) resulting in elevated cancer cell biosynthesis and secretion of acetylated polyamines (Fahrmann et al., 2020). We further reported that plasma polyamines, particularly DAS, are associated with TNBC development and progression (Fahrmann et al., 2020). Given our prior findings, we posit that the elevation in polyamines may underly an aggressive subtype of TNBC (Fahrmann et al., 2020) that is less likely to respond to NACT.

Elevated serum levels of urate, a purine catabolite, are also reported to be prognostic for TNBC recurrence and poor overall survival (Ackermann and Tardito, 2019; Gong et al., 2021). Lysophosphatidylethanolamines and lauroylcarnitine are associated with cancer metabolic plasticity and fatty acid oxidation (Melone et al., 2018). We have previously reported that JAK/STAT3-mediated fatty acid oxidation promotes chemoresistance in TNBC (Chakraborty et al., 2016; Wang et al., 2018). Methylhistidine has been shown to be elevated in serum of TNBC patients who had an cPR following NACT (He et al., 2021). TNBC cells are reported to exhibit a glutamine-dependent phenotype; promoting survival advantage as well as chemo-resistance (Kung et al., 2011; Lampa et al., 2017).

Remarkably, among the metabolites in the metabolite panel were two microbiome-related metabolites, indoleacrylic acid (IAA) and indole-acetylaldehyde (IAALD). IAA and IAALD are produced through the catabolism of tryptophan by the gut microbes (Vujkovic-Cvijin et al., 2013). Increasing evidence implicates that alterations in the microbiome influence resistance to anticancer treatment, including conventional chemotherapy, immunotherapy, radiotherapy, and surgery (Pryor et al., 2020; Garajová et al., 2021; Pandey and Umar, 2021). The relationship between changes in the microbiome and response to NACT warrants further investigation.

On balance, limitations to our study include limited sample availability and lack of external validation. To assess for potential overfitting, we tested the model by introducing perturbation (e.g., random selection and replacement) to the dataset and re-evaluated performance, the results of which demonstrated that our model was robust. We performed further validation using available samples and found that the metabolite panel provided good classifier performance for distinguishing individuals with RCB-II/III following four cycles of NACT from those with RCB-0/I, thus providing an independent validation. We note that attenuation of model performance after four cycles of NACT could be attributable to elevations in plasma metabolites consistent with chemotherapy-induced cancer cell death and turnover. Additionally, the relative cost-effectiveness of using the metabolite panel for risk-based prediction of non-responsiveness to NACT needs to be considered compared to other clinical predictors (Croshaw et al., 2011; Shin et al., 2011; Ono et al., 2012; De Los Santos et al., 2013; Leon-Ferre et al., 2018).

In conclusion, using a deep learning model, we developed a blood-based metabolite panel and that offers potential utility for identifying TNBC patients who are at high-risk of being non-responsive to NACT and who may benefit from more personalized treatment modalities.

## Data Availability Statement

The data presented in the study is available at the NIH Common Fund's National Metabolomics Data Repository (NMDR) website, the Metabolomics Workbench, where it has been assigned Study ID ST002235. The data can be accessed directly via its Project DOI: http://dx.doi.org/10.21228/M8HX4C.

## Ethics Statement

The studies involving human participants were reviewed and approved by patients with stage I–III TNBC enrolled in the prospective, Institutional Review Board (IRB)-approved, clinical study, A Robust TNBC Evaluation framework to Improve Survival (ARTEMIS, NCT02276443), were included in this study. Cancer-free women (*n* = 167) were obtained from the MD Anderson Cancer Center (MDACC) Longitudinal High-Risk Cohort initiated September 1st, 2011, for the prospective follow-up of cancer-free high-risk women seen in the MDACC Cancer Prevention Center (IRB protocol LAB07-0086). The patients/participants provided their written informed consent to participate in this study.

## Author Contributions

EI, SH, and JF: conceptualization. EI, RW, EM, JD, JLo, KD, and JF: methodology. EI and JF: validation, visualization, writing-original draft preparation, and formal analysis. ER, BL, JL, DT, VV, SD, GR, BAd, BAr, RC, JW, and AB: resources. RW, EM, JD, JW, and JF: data curation. RW, JV, EM, RS, JD, SM, ER, BL, JLi, DT, VV, SD, GR, BAd, RC, JW, AB, BAr, JLo, KD, and SH: writing-review and editing. SH and JF: supervision. JLo, KD, and SH: funding acquisition.

## Funding

The work presented was supported by Cancer Prevention & Research Institute of Texas (CPRIT) (RP180505) (SH), the generous philanthropic contributions to the University of Texas MD Anderson Cancer Center Moon Shots Program™, the Little Green Book Foundation, and the Still Water Foundation. KD was partially supported by a Cancer Center Support Grant NCI Grant P30 CA016672, NIH grants UL1TR003167, 5R01GM122775. JLo was partially supported by the National Cancer Institute and the National Center for Advancing Translational Sciences of the NIH (P30CA016672 and CCTS UL1TR003167).

## Conflict of Interest

Author SM declares honoraria from Novartis, Pfizer and research funding from Oncothyreon (Inst), Pfizer (Inst), Novartis (Inst), Genentech (Inst), Takeda (Inst), Bayer (Inst), EMD Serono (Inst), Genentech (Inst); travel, accommodations, expenses: Novartis, Pfizer. Author JLi declares consulting or advisory role: Pfizer, AstraZeneca, Medivation/Pfizer; speakers' bureau from Physician Education Resource, UpToDate, Med Learning, Group, Medscape; research funding from Novartis (Inst), Bristol-Myers Squibb (Inst), Genentech (Inst), Pfizer (Inst), EMD Serono (Inst), Jounce Therapeutics (Inst), GlaxoSmithKline (Inst), Medivation/Pfizer (Inst); patents, royalties, other intellectual property: UpToDate; travel, accommodations, expenses: Physician Education Resource, Med Learning Group, Medscape. Author DT declares consulting or advisory role: AstraZeneca, Genomic Health, Gilead Sciences Inc, GlaxoSmithKline, Novartis Pharma, OncoPep, Pfizer; research funding: Pfizer, Novartis Pharma. Author VV declares honoraria: Genentec; consulting or advisory role: Genentech; travel, accommodations, expenses: Genentech. Author SD declares honoraria: Novartis; consulting or advisory role: Tempus, Taiho Pharmaceutical, Pfizer; research funding: EMD Serono, Guardant Health; travel, accommodations; expenses: Phillips Gilmore Oncology Communications. Author BAd declares consulting or advisory role: Bright Pink, AbbVie; research funding: AbbVie (Inst), PharmaMar (Inst), AstraZeneca (Inst), InVitae (Inst); travel, accommodations, expenses: AstraZeneca. The remaining authors declare that the research was conducted in the absence of any commercial or financial relationships that could be construed as a potential conflict of interest.

## Publisher's Note

All claims expressed in this article are solely those of the authors and do not necessarily represent those of their affiliated organizations, or those of the publisher, the editors and the reviewers. Any product that may be evaluated in this article, or claim that may be made by its manufacturer, is not guaranteed or endorsed by the publisher.
