# Supplementary material for: Application of Artificial Intelligence to Plasma Metabolomics Profiles to Predict Response to Neoadjuvant Chemotherapy in Triple-Negative Breast Cancer
Source: Front Artif Intell. 2022 Aug 11;5:876100. doi: 10.3389/frai.2022.876100 (PMC9403735; doi:10.3389/frai.2022.876100)

**Supplementary Tables and Figures**

**Supplemental Table S1. Performance estimates of individual metabolites for distinguishing TNBC patients that had RCB-II/III following NACT compared to those that had RCB-0/I.**

**Supplementary Table S2**. **Feature importance of selected metabolites.**

**Supplementary Table S3. Predictive performance of the DLM for differentiating RCB-I/II/III from RCB-0.**

**Supplementary Table S4. Stability check of the DLM.**

**Supplementary Figure S1. Association between tumor stage and % Ki-67 tumor staining positivity with RCB status.**

**Supplementary Figure S2. Spearman correlation heatmap for selected metabolites.**

**Supplementary Table S1.**

*See Excel Dataset*

**Supplementary Table S2**. **Feature importance of selected metabolites.** Values represent importance scores for each metabolite calculated using the Gedeon method (*see methods*).

| **Variable** | **Relative Importance** |
| --- | --- |
| Lauroylcarnitine | 1.00 |
| Indoleacrylic acid | 0.92 |
| Methylhistidine | 0.81 |
| Urate | 0.76 |
| Diacetylspermine | 0.72 |
| Tyramine | 0.70 |
| Cholesterol glucuronide | 0.64 |
| Acetylspermidine | 0.63 |
| Indole-3-acetyaldehyde | 0.58 |
| Lysophosphatidylethanolamine(18:2) | 0.56 |
| Glutamine | 0.55 |

**Supplementary Table S3. Predictive performance of the DLM for differentiating RCB-I/II/III from RCB-0.**

| **Variable** | **DLM** |
| --- | --- |
| **RCB-0 (N)** | 48 |
| **RCB-I/II/III (N)** | 40 |
| **AUC (95% CI)** | 0.76 (0.65 - 0.87) |
| **Sensitivity @ 95% specificity** | 0.48 (0.12 - 0.70) |
| **Specificity @ 95% sensitivity** | 0.06 (0.00 - 0.29) |
| **AUC (95% CI) Stage I/II** | 0.76 (0.63 - 0.89) |
| **AUC (95% CI) Stage III** | 0.78 (0.53 - 1.00) |
| **Odds Ratio (95% CI)** | 4.54 (2.26 - 11.18) |
| **Adjusted Odds Ratio (95% CI)†** | 5.76 (2.55 - 17.30) |

† age and stage were included as covariables in adjusted odd ratios.

**Abbreviations: AUC**- Area under the Receiver Operating Characteristic Curve; **DLM-** deep learning model; **Cl-** confidence intervals; **N**- number of patients; **RCB**- residual cancer burden

**Supplementary Table S4. Stability check of the DLM.**

|  | **Scenario #1** | **Scenario #2** | **Scenario #3** | **Scenario #4** | **Scenario #5** |
| --- | --- | --- | --- | --- | --- |
| **Variable** | 500 samples (randomly selected) with replacement | 100 samples (randomly selected) with replacement | RCB-0 vs (RCB II and RCV III) | RCB-I vs (RCB II and RCV III) | (RCB-I and RCB-0) vs (RCB II) |
| **N0 (responder)** | 359 | 63 | 48 | 14 | 62 |
| **N1 (non-responder)** | 141 | 37 | 26 | 26 | 21 |
| **AUC (95% CI)** | 0.97 (0.96 - 0.98) | 0.95 (0.91 - 0.99) | 0.95 (0.91 - 1.00) | 1.00 (1.00 - 1.00) | 0.96 (0.93 - 1.00) |
| **Sensitivity @ 95% sp** | 0.88 (0.73 - 0.96) | 0.51 (0.22 - 0.97) | 0.73 (0.19 - 0.96) | 1.00 (1.00 - 1.00) | 0.81 (0.43 - 1.00) |
| **Specificity @ 95% sn** | 0.87 (0.82 - 0.96) | 0.83 (0.73 - 0.95) | 0.84 (0.67 - 0.98) | 1.00 (1.00 - 1.00) | 0.89 (0.76 - 0.98) |
| **AUC (95% CI) Stage I/II** | 0.98 (0.96 - 0.99) | 0.96 (0.92 - 1.00) | 0.96 (0.92 - 1.00) | 1.00 (1.00 - 1.00) | 0.97 (0.94 - 1.00) |
| **AUC (95% CI) Stage III** | 0.97 (0.94 - 1.00) | 0.96 (0.88 - 1.00) | 0.94 (0.81 - 1.00) | 1.00 (1.00 - 1.00) | 0.96 (0.86 - 1.00) |

**Abbreviations: AUC**- Area under the Receiver Operating Characteristic Curve; **DLM-** deep learning model; **Cl-** confidence intervals; **RCB**- residual cancer burden; **sp**- specificity; **sn**- sensitivity

**Supplementary Figure S1. Association between tumor stage and % Ki-67 tumor staining positivity with RCB status. A)** Contingency table shows the # of TNBC cases stratified by stage (I/II vs III) that had RCB-0/I or RCB-II/III following NACT. **B)** Distribution plot illustrating pre-treatment % tumoral Ki-67 staining stratified based on whether the patient had an RCB-0/I or RCB-II/III status following NACT. P-values were determined by 2-sided Wilcoxon Rank Sum Tests.

**
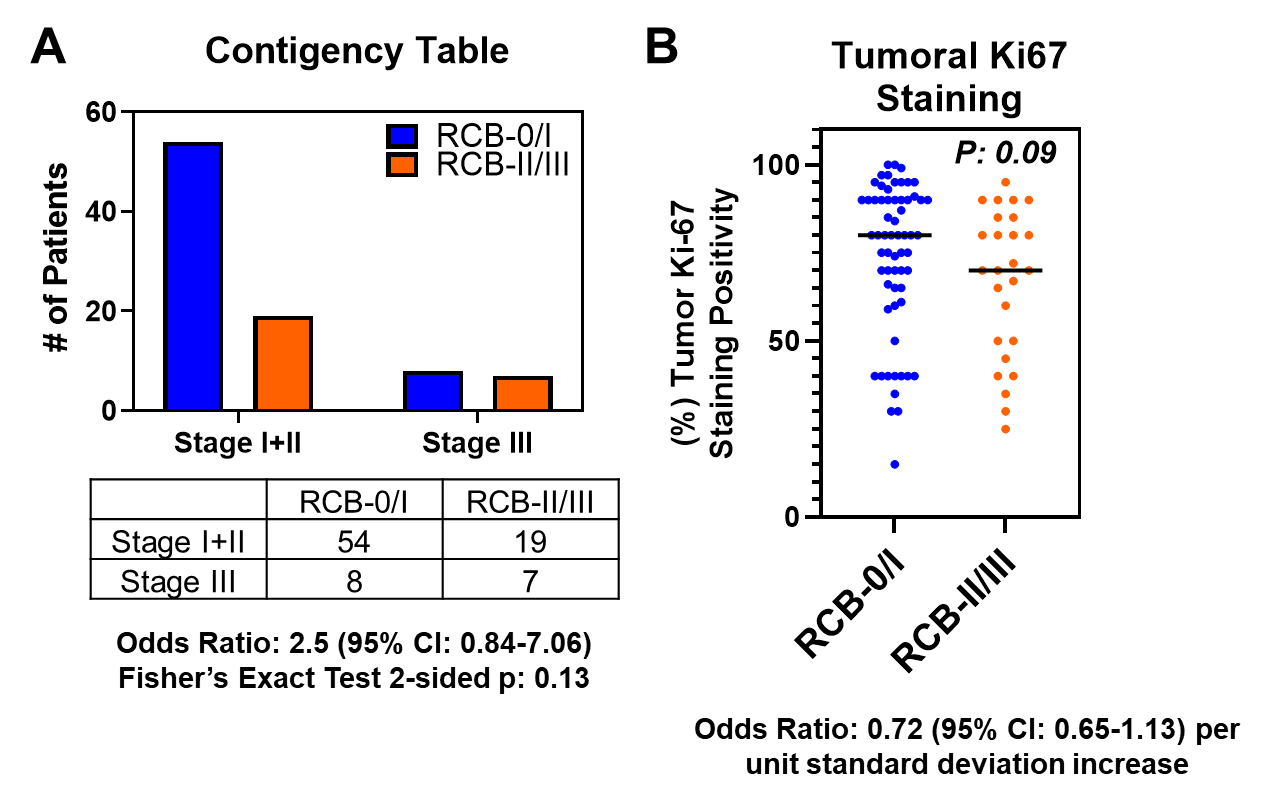
**

**Supplementary Figure S2. Spearman correlation heatmap for selected metabolites.**


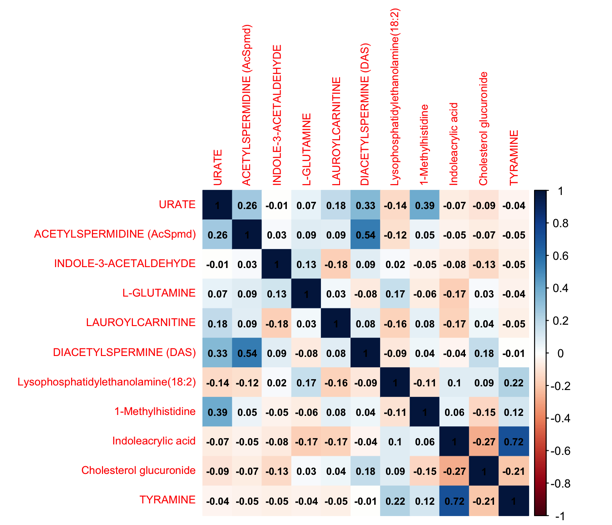

Supplement: Supplementary file 2 [file Data_Sheet_1.docx]
